# X-ray microtomography (microCT) of male genitalia of *Nothybus kuznetsovorum* (Nothybidae) and *Cothornobata* sp. (Micropezidae)

**DOI:** 10.3897/zookeys.744.22347

**Published:** 2018-03-20

**Authors:** Tatiana V. Galinskaya, Dina Gafurova (Gilyazetdinova), Olga G. Ovtshinnikova

**Affiliations:** 1 Faculty of Biology, Lomonosov Moscow State University, Moscow, 119234 Russia; 2 Museum of Entomology, All-Russian Plant Quarantine Center, Pogranichnaya 32, Bykovo, 140150, Russia; 3 Faculty of Geology, Lomonosov Moscow State University, Moscow, 119234 Russia; 4 Zoological Institute, Russian Academy of Sciences, St. Petersburg, 199034 Russia

**Keywords:** *Cothornobata*, Morphology, musculature, *Neria commutata*, *Nothybus kuznetsovorum*, sclerites

## Abstract

The results of manual dissection of the musculature of the male genitalia in *Nothybus kuznetsovorum* are fully confirmed by the modern methods of Micro-CT. A comparative analysis of *Neria commutata* and *Cothornobata* sp. shows that an increase in the flexion in the genitalia of males and the displacement of syntergosternite VII to the ventral side in *Cothornobata* sp. caused the disappearance of the muscles ITM6–7r and ITM7–8r. In addition, this increase in flexion apparently caused the fusion of the M18 muscles into one bundle. The muscle ISM5-6c goes on to moving the second segment of the forcipate appendages of sternite V.

## Introduction

Micropezidae is an average-sized family of acalyptrate flies (Diptera). It comprises approximately 700 described species in 50 genera (Marshall 2010, 2012; Ferro and Carvalho 2014). The family is globally distributed, with the greatest species richness found in tropical regions (Steyskal 1968). The body of adult flies is 3.5 to 20.0 mm long (Steyskal 1987).

The family Nothybidae includes only one genus, *Nothybus* Rondani, 1875, distributed in the Oriental Region. These acalyptrate flies are 5.5 to 15.0 mm body long (Lonsdale and Marshall 2016).

Study of the musculature is helpful not only for specifying the functions of genital sclerites, but also for revealing the homology of some poorly traced structures (Ovtshinnikova 1989, 1994, Ovtshinnikova and Yeates 1998; Galinskaya and Ovtshinnikova 2015).

Previously specimens of *Nothybus kuznetsovorum* Galinskaya et Shatalkin, 2015 (Nothybidae) and *Neria commutata* (Czerny, 1930) (Micropezidae) were investigated by manual dissection using light microscopy (Galinskaya et al. 2016, Ovtshinnikova and Galinskaya 2017). In these studies, some muscles were thin and indistinct.

Among the Diptera the X-ray micro-computed tomography (Micro-CT) was used for revealing the morphology of the feeding apparatus of *Philoliche rostrata* and *Ph. gulosa* (Tabanidae) (Karolyi et al. 2014); for the characterization of the morphology of the venom system of *Eutolmus rufibarbis* (Asilidae) (Drukewitz et al. 2018); for revealing the morphology of the proboscis, the food canal and suction pump muscles of *Prosoeca* sp. (Nemestrinidae) (Karolyi et al. 2013); for analysis of the *Drosophila* larvae central nervous system (Drosophilidae) (Mizutani et al. 2007); for revealing the morphology of the late pupa of *Calliphora vicina* (Calliphoridae) (Metscher 2009); for performing qualitative and quantitative analyses of the morphological changes taking place during the intra-puparial period of *Calliphora vicina* and *Lucilia sericata* (Calliphoridae) (Richards et al. 2012; Hall et al. 2017; Martín-Vega et al. 2016; Martín-Vega et al. 2017a, b); and for the visualization of the three-dimensional movements of flight muscles and thoracic sclerites of *Calliphora vicina* (Calliphoridae) (Walker et al. 2014).

In this study X-ray micro-computed tomography (micro-CT) was utilizzed for revealing genital sclerites and muscles of *Nothybus kuznetsovorum* (Nothybidae), comparing it with results of manual dissection (Galinskaya et al. 2016). The sclerites and muscles of *Cothornobata* sp. (Micropezidae) are also revealed. Males of Micropezidae are characterized by specialized forceps-like processes of variable size, shape, and ornamentation arising from sternite V and by sternite VI sometimes with specialized processes (these processes are secondarily reduced in some spp). The examined species is characterized by elongated forcipate appendages of sternite V, and the musculature of these appendages was investigated.

## Materials and methods

One specimen of *Nothybus kuznetsovorum* and one specimen of *Cothornobata* sp. were collected in Northern Vietnam, PhuTho Province, Thanh Son District, Xuan Son National Park; 21°8.333'N; 104°56.25'W; h = 300–900 m, 23 October 2014 by T.V. Galinskaya. The specimens were fixed in 70% ethanol. They were prepared for X-ray micro-computed tomography (micro-CT) analysis by contrasting with iodine as outlined in Gignac and Kley (2014) and by critical point drying. Specimens were glued with the thorax pointing downwards on the tip of a small pin of 1 mm diameter, with the tip of the male abdomen as close to the rotation axis as possible. Micro-CT scans were produced under phase contrast (40 KV, 8 W), using a 4× detector (10 s; 4.15 μm pixel size) and 10× detector (30 s; 1.89 μm pixel size).

The male genital muscles were classified into several groups (muscles of the epandrial complex, muscles of the hypandrial complex, tergosternal muscles, and pregenital muscles) and described according to the system of Ovtshinnikova and Galinskaya (Ovtshinnikova 1989, 2000; Ovtshinnikova and Galinskaya 2017). Male genital sclerites were described according to the Sinclair (2000).

## Results

### *Nothybus kuznetsovorum* Galinskaya & Shatalkin, 2015

Figure 1

**Figure 1.**
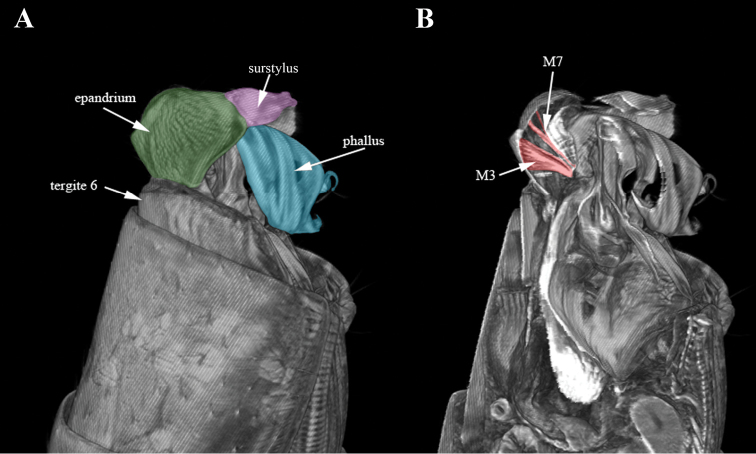
Micro-CT surface rendering (**A**) and volume rendering of virtual sections to median, digitally stained (**B**) of *Nothybus kuznetsovorum* (Nothybidae), lateral view. Cerci shown in yellow, epandrium in dark green, phallus in light blue, surstylus in pink, syntergosternite VIII in violet, syntergosternite VII in light green, sternite VI in orange and sternite V in dark blue.

All sclerites and muscles and their places of attachments revealed using manual dissection and decribed by us previously (Galinskaya et al. 2016) have been confirmed by micro-CT. The only questionable muscle after manual dissection was M7 (Galinskaya et al. 2016). Here the presence and attachment sites of attachment of M7 are confirmed (Figure 1B).

### *Cothornobata* sp.

Figures 2–4

**Sclerites.** Sternite IV elongate. Sternite V modified into elongated forcipate appendages. Sternite VI elongate. Tergite IV, V, VI not modificated. Ejaculatory apodeme placed at the level of sternites V and VI. Sternite and tergite VII fused into syntergosternite positioned on left side of body. Syntergosternite VIII spherical. Epandrium large, bearing bifurcate surstyli and small cerci.

**Muscles.** The muscles are grouped by the site of insertion of their proximal parts. Thin paired muscles ISM4–5 attached to basal area of sternite V (Figure 3B). Tergosternal muscle TSM4 poorly developed (Figure 4A).

Muscles of segments V and VI. Two pairs of muscles ISM5–6 lying between sternites V and VI: proximal retractors of sternite VI ISM5–6a broadly fan-shaped, extending from basal area of sternite V to lateral surface of sternite VI (Figures 3C, 4C); median flexors of forcipate appendages ISM5–6c broadly fan-shaped, extending from lateral outgrowths of sternite V to central part of sternite VI (Figures 3B, 4B, G). Contraction of muscles ISM5–6 powers the forcipate appendages of sternite V that participate in the fixation of the female’s abdomen during copulation. Tergosternal muscles TSM5 and TSM6 in segments V–VI poorly developed (Figure 4A).

Muscles associated with segment VII are asymmetrical. Left intersegmental tergal muscle ITM6–7l wide, extending from distal area of tergite VI to left lateral margin of syntergosternite VII (Figure 4G). No muscles TSM6–7 present in sternal area between segments VI and VII. Left muscle ISM7–8l wide, extending from basal margin of syntergosternite VII to syntergosternite VIII (Figure 4G). Tergosternal muscles of segment VII absent, probably due to fusions or reductions.

Pregenital muscles. Muscle of hypandrium M18 connects syntergosternite VIII to hypandrium (Figure 4G). Retractors of epandrium M19 extending from syntergosternite VIII to middle of proximal margin of epandrium (Figure 4C, F).

Tergosternal muscles of segment VIII absent, probably due to fusions or reductions.

Long and powerful symmetrical tergosternal abductors M5 extending from latero-basal margin of epandrium to latero-distal margin of hypandrium.

**Figure 2.**
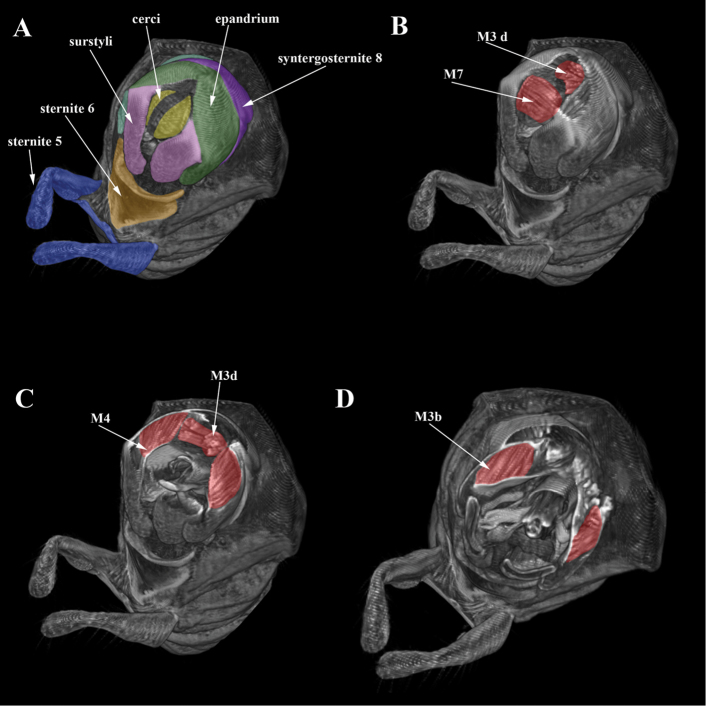
Micro-CT surface rendering (**A**) and volume rendering of virtual sections posteriorly, digitally stained (**B–D**) of *Cothornobata* sp. (Micropezidae), posterior view.

Muscles of epandrial complex symmetrical. Wide and thin paired cercal muscles M7 extend from distal outgrowths of subepandrial sclerite to cerci (Figure 2B). Paired muscles of subepandrial sclerite M3 well developed, comprise two pairs of muscles: muscles M3b extending from latero-distal lobes of subepandrial sclerite to latero-distal part of epandrium (Figure 2D, 4B, E); muscles M3d extending from median bridge of subepandrial sclerite to distal part of epandrium (Figure 2B, C). Short and narrow adductors of surstylus M4 connecting laterodistal parts of epandrium to basal part of surstylus (Figure 2C, 4B).

Muscles of hypandrial complex symmetrical. Short and powerful paired phallic retractors M1 connecting distal part of phallapodeme to distal inner part of hypandrium (Figure 4C). Long and powerful paired phallic protractors M2 connecting distal part of phallapodeme to lateral and median parts of hypandrium (Figure 4B) and lying above M1. Paired retractors of pregonites M42 extending from inner distal part of hypandrium to pregonites (Figure 4B). Compressors of ejaculator M23 well developed (Figure 4G).

**Figure 3.**
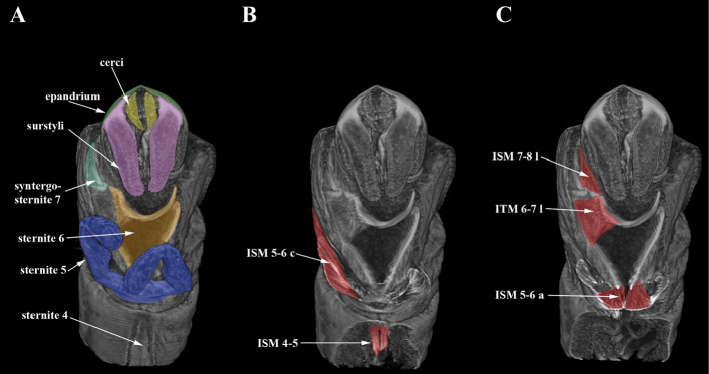
Micro-CT surface rendering (**A**) and volume rendering of virtual sections right to median, digitally stained (**B–C**) of *Cothornobata* sp. (Micropezidae), abdominal view.

**Figure 4.**
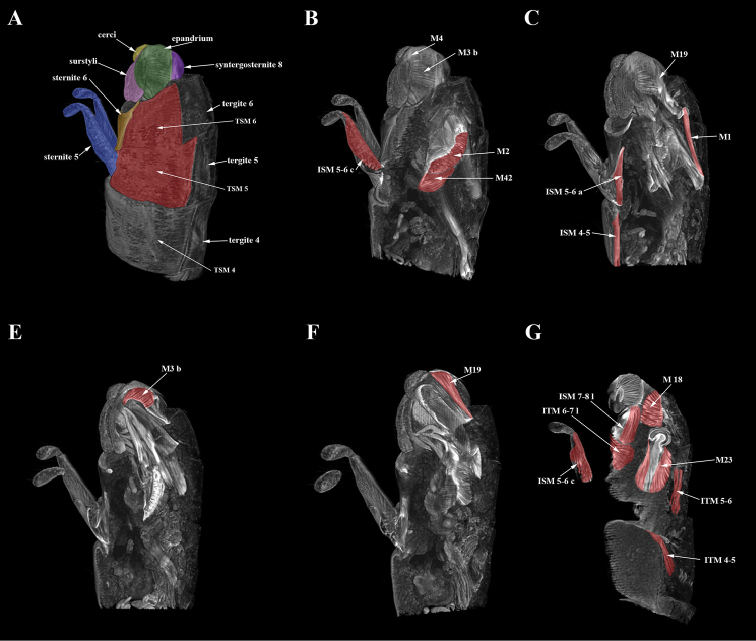
Micro-CT surface rendering **(A**) and volume rendering of virtual sections right to median, digitally stained (**B–G**) of *Cothornobata* sp. (Micropezidae), lateral view.

## Discussion

Since the results of the study of the male genitalia in *Nothybus kuznetsovorum* using micro-CT completely coincide with the results of manual dissection, we conclude that the method of manual anatomy has not lost its significance. However, the micro-CT takes much more time than manual anatomy. The undoubted advantage of micro-CT is its higher accuracy and the fact that only one specimen is needed for the study, while manual anatomy usually needs 4–5 specimens.

We assume that the presence of the elongated appendages of sternite V in the species *Cothornobata* sp. can cause the presence of additional muscles ISM5–6 (due to the complication of sclerites in *Cothornobata* sp. comparing with *Neria commutata*). However, no muscle going from the basal to the distal segments of the appendages of V sternite was discovered. Additionally, we did not find the muscles ISM5–6b and ISM5–6d. *Neria commutata* has the lateral flexors of forcipate appendages ISM5–6d broad and short, extending from inner vanes of sternite V to those of sternite VI and occupying a considerable part of the surfaces of both sclerites; the distal retractors of sternite VI ISM5–6b are narrow, extending from the distal parts of the outgrowths of sternite V to the distal area of sternite VI. Apparently, the muscle ISM5–6c goes on to moving the distal half of the forcipate appendages of sternite V.

In *Cothornobata* sp., unlike *Neria commutata*, no muscles ITM6–7r and ITM7–8r have been detected. *Neria commutata* has the right muscle ITM6–7r narrow, conical, extending from the median part of tergite VI to the membrane in front of syntergosternite VII, and the right muscle ISM7–8r small and short, extending from the membrane at syntergosternite VII to syntergosternite VIII (Ovtshinnikova and Galinskaya 2017). We assume that in *Cothornobata* sp. these muscles are absent due to a stronger flexion of syntergosternite VII to the ventral side. Increased flexion of syntergosternite VII in *Cothornobata* sp. caused the disappearance of muscles ITM6–7r and ITM7–8r. Also, an increase of the flexion apparently caused the fusion of the M18 muscles into one bundle.
